# A Case Report on BRASH (Bradycardia, Renal Failure, Atrioventricular Blockade, Shock, and Hyperkalaemia) Syndrome: A Challenging Diagnosis

**DOI:** 10.7759/cureus.32704

**Published:** 2022-12-19

**Authors:** Pooja Roy, Zahin Islam Rafa, Harshita Agrawal, Soumyadipto B Arko

**Affiliations:** 1 Internal Medicine, Harlem Hospital Center, New York, USA; 2 Internal Medicine, Ibn Sina Medical College Hospital, Dhaka, BGD; 3 Laboratory Medicine and Pathology, Mayo Clinic, Rochester, USA; 4 Medicine, Dhaka Medical College Hospital, Dhaka, BGD

**Keywords:** brash syndrome, cough, shock, renal failure, hyperkalemia, bradycardia

## Abstract

A relatively new yet critical phenomenon of bradycardia, renal failure, atrioventricular (AV) blockade, shock, and hyperkalemia (BRASH) syndrome is hypothesized to happen in patients who take atrioventricular nodal blocking (AVNB) agents and have underlying renal insufficiency. In our case, a 67-year-old female with an extensive medical history presented to the emergency room with chief complaints of decreased appetite, nausea, vomiting, fatigue, and left-sided atypical chest pain for the past two weeks. The patient was taking losartan potassium 50 mg daily in addition to carvedilol 6.25 mg twice daily for her hypertension (HTN) and heart failure with reduced ejection fraction (HFrEF) with the addition of bumetanide 0.5 mg, which was added three weeks prior. On presentation, the patient had sinus bradycardia and hypotension along with the laboratory finding of acute kidney injury (AKI) in the setting of chronic kidney disease (CKD) and hyperkalemia. Cardiology and nephrology were consulted emergently; her clinical scenario raised suspicion of the BRASH syndrome. The patient was admitted to the intensive care unit (ICU), and all antihypertensive medications, including beta-blockers, were stopped. Intravenous (IV) fluid resuscitation and medical management of hyperkalemia were initiated, along with BiPAP for respiratory distress. She responded significantly, and her vitals remained stable. She was successfully discharged home with a cardiology and nephrology follow-up. We highlight the case to emphasize the consideration of BRASH in a patient on multiple cardiac medications who presented with deranged electrolytes and organ failure, and decompensated heart failure (HF) should not be fixed on as the principal diagnosis.

## Introduction

Joshua D. Farkas observed a phenomenon in 2016 in which a combination of hyperkalemia and AV nodal blockade led to profound bradycardia in patients with secondary renal insufficiency. The phenomenon is now known as the "BRASH phenomenon" or "BRASH syndrome" [1]. This novel, under-recognized clinical entity is epitomized by a clinical quintet of bradycardia, renal failure, atrioventricular (AV) nodal blockade, shock, and hyperkalemia (BRASH) [1-7]. Although the incidence of BRASH syndrome is unknown, it is feasible for this uncommon manifestation to present in the emergency department due to the widespread use of atrioventricular (AV) nodal blockers for hypertension, chronic renal disease, and ischemic heart disease [2]. So far, there have been limited studies that have defined the demographics, definitive clinical features, severity of the illness, or prognosis of BRASH syndrome [3]. However, this is more frequent in older adults with heart disease and low renal reserve owing to the use of antihypertensive medications and having borderline renal function [4].

The existing literature available on BRASH syndrome has a great deal of variation in terms of clinical presentations, contributing variables, and therapy [3,5,6]. Although the incidence of each symptom is not well reported, preliminary symptoms may include generalized weakness or nonspecific symptoms like syncope and can manifest in a variety of ways, from coincidental bradycardia to multiorgan failure [5]. The prevalence of BRASH syndrome is greater than what has been documented in the medical literature. When a patient with modest hyperkalemia develops substantial bradycardia, emergency doctors should have a high degree of suspicion for BRASH syndrome [1,3,4].

Primarily, even if it's only minor, hyperkalemia has to be treated very quickly. As is the case with severe hyperkalemia, therapy should begin with intravenous delivery of insulin, glucose, and calcium for membrane stability. Because IV calcium is not used in the advanced cardiac life support (ACLS) bradycardia protocol, BRASH syndrome is not properly treated. Thus, excessive transvenous pacing may result from naively following the ACLS bradycardia protocol without appropriate knowledge of the BRASH syndrome [4,5]. According to one PRISMA study, which included 34 articles comprised of one observational study, 18 case reports and series, and 15 conference abstracts revealed that patients' symptoms could not be relieved by atropine or glucagon; 59.5% needed inotropes or chronotropes instead, and 7.1% of people died due to BRASH syndrome [3].

Here, we describe the case of a patient who presented with chest pain, vomiting, orthopnea, epigastric pain, and cough, and was ultimately diagnosed and managed as having BRASH syndrome. 

## Case presentation

A 67-year-old African American woman with a past medical history (PMH) of severe coronary artery disease (CAD) status post (S/P) coronary artery bypass graft (CABG x 2), with left internal mammary artery (LIMA) to the left anterior descending artery (LAD) and saphenous vein graft (SVG) to the obtuse marginal (OM) in 2019, type 2 diabetes mellitus (T2DM) and diabetes mellitus (DM) peripheral neuropathy, hypertension (HTN), heart failure with reduced ejection fraction (HFrEF), CKD S3aA1 came to the emergency department (ED) due to a loss of appetite, nausea and vomiting, fatigue, and left-sided chest pain for the past two weeks. The patient endorsed non-specific left-sided chest pain that radiated to the right side (7/10 intensity) but was not present during the evaluation. She also had two episodes of non-projectile, non-bilious, and non-bloody vomiting. She had epigastric pain of 7/10 intensity, bloating, reduced appetite, three-pillow orthopnea, a nonproductive cough, and contact with coughing grandchildren, but she denied shortness of breath, paroxysmal nocturnal dyspnea, fever, chills, or recent travels. She visited her primary care physician (PCP) three weeks ago for pedal edema and was prescribed bumetanide 0.5 mg daily. She was also taking losartan potassium 50 mg daily for her BP and carvedilol 6.25 mg twice daily for her hypertension (HTN) and heart failure with reduced ejection fraction (HFrEF).

At the ED, her vitals were as follows: blood pressure was 84/53 mmHg, heart rate was 56 beats per minute, and respiratory rate was 16 breaths per minute. She was saturating well in room air (RA). On systemic examination, there were mild left-sided basal crepitations and bilateral trace pedal edema; however, no jugular venous distension (JVD) was noted. The point-of-care ultrasound (POCUS) showed a collapsed inferior vena cava (IVC). A complete blood count (CBC) revealed anemia of chronic disease with hemoglobin/hematocrit of 10.1 g/dL/29.2%, white blood cells (WBC) of 10.41*103/mcL, and platelets of 147*103/mcL. Her basic metabolic panel (BMP) showed hyponatremia, hyperkalemia, hypochloremia, and acute kidney injury on CKD; sodium was 125 mmol/L, potassium was 5.9 mmol/L, chloride was 88 mmol/L, and blood urea nitrogen (BUN) and creatinine (Cr) were 96 mg/dl and 3.4 mg/dl, respectively (baseline Cr of 1.14 mg/dl). Venous blood gas (VBG) showed high anion gap metabolic acidosis with elevated lactate: anion gap was 21, pH was 7.36, PCO2 was 23 mmHg, PO2 was 86 mmHg, HCO3 was 13 mmol/L, and lactate was 5.5 mmol/L. Her proBNP was elevated to 41,385 (baseline 8k) (Table 1).

**Table 1:** Table displaying important values during admission, interval, and discharge. CO2: carbon dioxide; BUN: blood urea nitrogen; eGFR: estimated glomerular filtration rate

Laboratory values from admission, interval, and discharge with institution ranges provided				
Component	Latest reference range and units	10/3/2022	10/7/2022	10/11/2022
Sodium	136-145 mmol/L	125	122	132
Potassium	3.5-5.1 mmol/L	5.9	4.7	4.4
Chloride	98-107 mmol/L	88	89	96
CO2	22-29 mmol/L	13	17	23
BUN	7-18 mg/dl	96	67	53
Creatinine	0.5-0.9 mg/dl	3.4	2.1	1.47
Glucose	74-109 mg/dl	225	101	78
Calcium	8.5-10.1 mg/dl	8.5	8.4	8.5
Anion gap	6-18 mEq/L	19	16	13
Osmolarity calc	275- 295 mOsm/L	279	265	278
eGFR(cr)	>=60 ml/min/1.73m2	14	25	34

**Figure 1:**
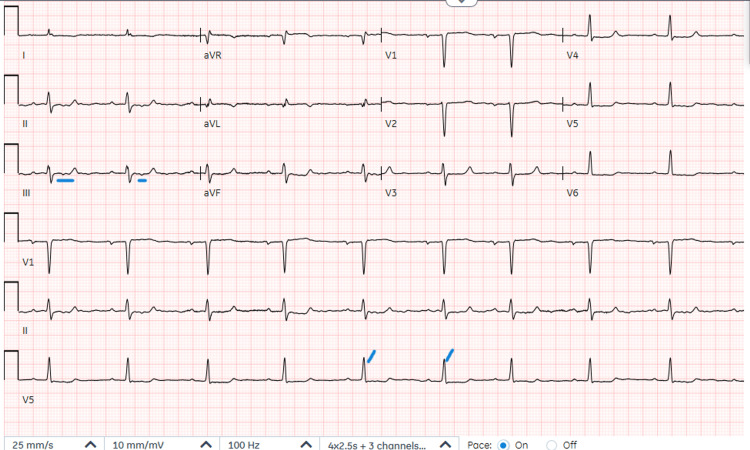
EKG demonstrating sinus bradycardia (a heart rate of 56 bpm indicated by the R-R interval in the V5 lead) and nonspecific ST-T wave abnormalities (indicated by a blue line in lead three).

Her chest X-ray showed small bilateral layering pleural effusions; cardiomegaly with acute and chronic pulmonary venous congestion; and a coronary artery bypass graft (CABG). The electrocardiogram (EKG) interpretation was sinus bradycardia, nonspecific ST-segment, and T-wave (ST-T) abnormalities (Figure 1). An ECHO was done to assess the cardiac condition, revealing a mildly dilated left ventricle with mildly increased wall thickness and paradoxical septal motion consistent with post-operative status, with the further demonstration of the left ventricle ejection fraction of 35%-40%, which was unchanged from her previous ECHO (video 1). In the setting of clinical and laboratory findings, her decompensated heart failure (HF) as part of the differential diagnosis was ruled out because she was not hypervolemic or fluid-overloaded. 

**Video 1:** ECHO showed a mildly dilated ventricle with mildly increased wall thickness and moderate LV diastolic dysfunction at 35%–40%. ECHO: echocardiogram; LV: left ventricle

Her admission matched the BRASH diagnostic criteria: bradycardia, renal failure, atrioventricular blockade, shock, and hyperkalemia metabolic acidosis while on beta blockers and angiotensin receptor blockers (ARBs). She was started with a sodium chloride 0.9% infusion and an electrolyte management hyperkalemia cocktail, including calcium gluconate (2 g), lokelma (10 g), insulin (5 units), D-50W 50%, albuterol (5 mg) via nebulizer (nebulized three times), and furosemide 40 mg via IV (4 times). Although her HCO3 was low, her PH remained > 7.20, and she did not require a bicarbonate infusion. Her home medications, beta-blockers, and ARB were discontinued in the interim. She responded well to the fluid resuscitation, as her BP improved and her physical condition improved with the correction of electrolyte and renal function.

**Figure 2:**
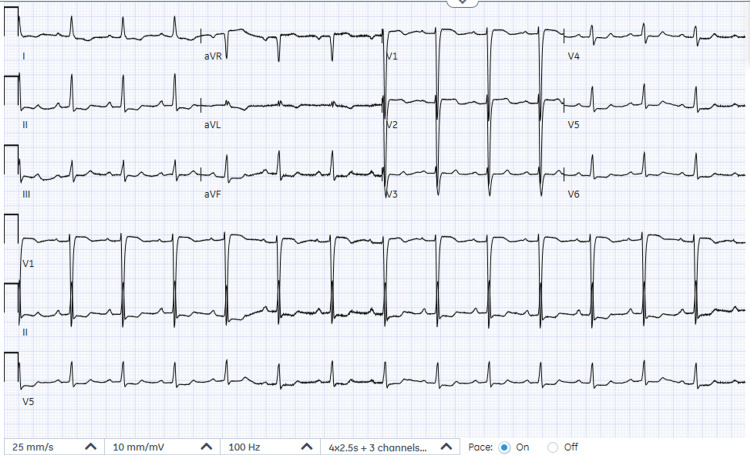
EKG showing sinus rhythm on discharge.

The patient was discharged after nine days of hospital admission for an outpatient cardiology and nephrology follow-up, with the recommendation to avoid angiotensin-converting enzyme (ACE) or angiotensin II receptor blockers (ARB) with concomitant use of beta-blockers. On discharge, the ECG revealed a normal sinus rhythm (Figure 2). Her discharge serum creatinine was 1.47 mg/dL, and her potassium was 4.4 mmol/L (Table 1).

## Discussion

The synergistic effects of AV nodal blockers and renal impairment can produce catastrophic bradycardia and hyperkalemia in BRASH syndrome, whose name is derived from the symptoms it generates: bradycardia, renal failure, AV nodal blockade, shock, and hyperkalemia. Despite the term being new, the link between these drugs and renal failure has long been known [6,8,9-12].

**Figure 3:**
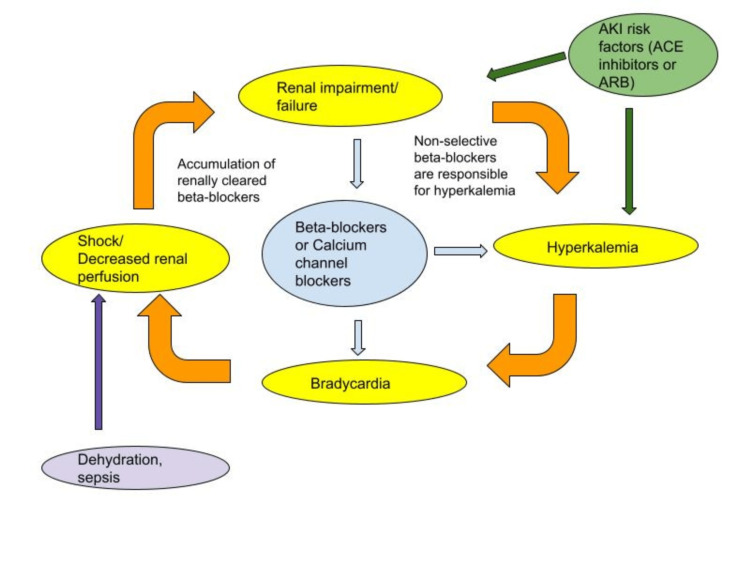
Vicious cycle of BRASH syndrome This illustration was made by the authors based on the source [1].

The vicious cycle of certain medications, hyperkalemia, and renal failure is the leading cause of this syndrome [1,2,5,13]. Hyperkalemia is driven by renal failure and may culminate in the buildup of certain AV node blockers (e.g., atenolol and nadolol). Beta-blockers cause hyperkalemia by suppressing aldosterone release from the adrenal cortex and reducing potassium uptake by cells [13]. ACE inhibitors' mode of action directly causes hyperkalemia. Aldosterone is not secreted downstream when angiotensin II is blocked. As a result, potassium and protons are discharged into the urine [13,14]. Due to the lower transmembrane potassium concentration, hyperkalemia causes the resting membrane potential (RMP) to become less negative and has two notable effects: it moves the RMP closer to the threshold and accelerates potassium efflux via accelerating phase three repolarization [13]. The myocardium becomes hypoexcitable when the RMP approaches the threshold, which decreases sodium input and lowers the rate of rise and the voltage of phase 0 of the action potential [13]. As a result of these factors (wide QRS, longer PR interval), the action potential's speed of propagation across the myocardium is slowed down, resulting in bradycardia [5,13]. Decreased heart rate decreases cardiac output, and this decreases renal perfusion, which causes renal impairment and eventually results in renal failure [1,5,13]. Renal failure further exacerbates hyperkalemia and causes AV nodal-blocking medication to accumulate due to decreased renal clearance [1,13]. Thus, the vicious cycle of the BRASH syndrome continues [13]. The potential of hyperkalemia to work in sync with AV node blockers to generate bradycardia is hypothesized to be the pathophysiologic key to BRASH syndrome (Figure 3).

Antihypertensives, acute renal damage, and dehydration are a few possible causes of BRASH syndrome. However, hypovolemia is evident in frequent cases and is considered a triggering factor too [7].

One of the numerous potential causes of reduced perfusion that might result in an acute kidney injury (serum creatinine by 0.3 mg/dL from baseline) is hypovolaemia, which can be brought on by either decreased intake or increased loss [3]. A hyperkalemic condition (K+ level of 5.5 mg/dL) results from reduced potassium excretion caused by acute renal injury. The body’s AV-blocking medications are considered to work synergistically with the hyperkalemic state to exacerbate bradycardia (HR < 60 beats/min), further reducing perfusion and exacerbating kidney injury [7].

BRASH syndrome represents an overlap between hyperkalemia and AV node blocker intoxication [4]. Therefore, it is important to distinguish between BRASH and pure hyperkalemia, as well as AV node blocker intoxication. Some distinguishing features include the following:

Patients with BRASH syndrome may only have modest hyperkalemia, and the AV node blocker and hyperkalemia work together to cause bradycardia [4]. Contrarily, bradycardia brought on by hyperkalemia alone typically necessitates a more marked increase in the potassium level. Bradycardia without additional hyperkalemia-related EKG characteristics (such as QRS widening or peaked T-waves) may favor BRASH syndrome [1].

Beta-blocker or calcium channel blocker intoxication can cause bradycardia and shock. The clinical history in comparison to BRASH syndrome may be the most crucial distinguishing aspect [1,3]. Most patients with BRASH syndrome take their medications as prescribed. The problem arises from a synergy between therapeutic drug levels and hyperkalemia rather than involving supratherapeutic drug levels [1,7]. Hyperkalemia and a significant clinical response upon intravenous calcium delivery are additional characteristics that may favor BRASH syndrome [1,3,6]. Patients using numerous AV nodal-blocking drugs to treat atrial fibrillation may be especially at risk [5,8,9]. Metoprolol and amlodipine, which are renally cleared AV nodal blockers, represent a higher risk because the concentration of the renal tubules drops as the GFR increases in an opposite manner. When exposed to dehydration or worsening renal function, elderly patients with a baseline impaired renal reserve and a cardiovascular illness controlled by beta blockers or calcium channel blockers are at risk of developing the BRASH syndrome. Other medications, such as ACE inhibitors, potassium-sparing diuretics, or nephrotoxic drugs, may be involved [10]. Even drugs like amiodarone or octreotide have also been documented to be linked to the disease’s development [3]. Up-titrating medicines that affect cardiac output or renal perfusion can also precipitate this [4].

Some drug interactions might facilitate BRASH syndrome, such as calcium channel blockers, nephrotoxins, or potassium-sparing diuretics such as spironolactone [1]. Angiotensin-converting enzyme inhibitors or angiotensin-receptor blockers can increase the risk of both hyperkalemia and renal dysfunction, as does digitalis [1,6]. Several beta-blockers, like atenolol and nadolol, are removed by the kidneys, which makes their levels rise during BRASH syndrome. Finally, nonspecific beta-blockers (e.g., labetalol) may promote hyperkalemia [1].

The most frequent early symptom of BRASH syndrome appears to be syncope [9]. According to a recent clinical analysis, 13 of 23 patients (56.5%) had presyncope or syncope, whereas the other patients had dyspnea, diaphoresis, chest pain, weakness, or lethargy either alone or in combination with presyncope or syncope [8,14].

The cornerstone of management is addressing all the individual factors that lead to this vicious cycle, including discontinuation of the offending medications, potassium shifting and elimination (e.g., administration of insulin or glucose, diuretics, potassium binders, and sodium bicarbonate in acidosis), reversal of kidney injury, calcium gluconate for cardiac membrane stabilization, and vasopressors. For patients with profound bradycardia, inotropes such as epinephrine or isoproterenol may be used along with critical treatment modalities like a temporary pacemaker [11,13,14]. Potassium-lowering treatment takes time to work [11]. Kaliuresis with potassium-wasting diuretics, such as furosemide, can be considered to facilitate the urinary excretion of potassium. Emergency dialysis should be given if the above measure fails. Transvenous pacing may be necessary but is typically used only as a salvage maneuver when the aforementioned treatments fail [1].

The propensity of patients with CKD to develop hyperkalemia warrants a meticulous review of concurrent medications. Atrioventricular-nodal blocking (AVNB) agents should not be the primary antihypertensive agents in patients with worsening chronic kidney disease (CKD). Additionally, it is important to recognize that any AVNB, including beta blockers and verapamil, can cause BRASH syndrome in patients with CKD in the setting of hyperkalemia [12].

If left untreated, BRASH syndrome can cause renal failure, and severe hyperkalemia can cause cardiac arrhythmias like ventricular fibrillation and asystole and eventually result in cardiac arrest [1,14]. Regrettably, the most frequent blunder in treating patients with BRASH syndrome is concentrating solely on one aspect (e.g., hyperkalemia), which leads to the neglect of other manifestations of this disease (e.g., renal failure, AV node blockers, and bradycardia). Acknowledging its pathogenesis is essential for providing a comprehensive treatment plan that addresses all of its elements [5]. The BRASH syndrome cycle can be broken with early aggressive therapy using beta-agonists, quick hyperkalemic therapies, and hemodynamic support. Once the triggering events are under control, patients typically have a remarkable improvement in heart function [2,5,10].

## Conclusions

The BRASH syndrome highlights the synergetic effect of mild hyperkalemia and beta-blockers/calcium channel blockers on their AV nodal blockage properties. Beta-blockers and ACEi/ARBs are essential medications for patients with heart failure with reduced ejection fraction (EF). The BRASH syndrome should not dissuade providers from prescribing these medications but make them vigilant in monitoring renal function and electrolytes, especially in patients on combination medications and borderline renal function. Though the pathophysiology is yet to be analyzed, the early recognition and evaluation in patients with suspicion of BRASH can prevent premature cardiac intervention and deterioration of the clinical condition. A proper medication history analysis and knowledge of BRASH syndrome can initiate the proper management and recovery of the patient.
